# Cannabis Use in Patients with Fibromyalgia: Effect on Symptoms Relief and Health-Related Quality of Life

**DOI:** 10.1371/journal.pone.0018440

**Published:** 2011-04-21

**Authors:** Jimena Fiz, Marta Durán, Dolors Capellà, Jordi Carbonell, Magí Farré

**Affiliations:** 1 Human Pharmacology and Neurosciences Unit, Institut de Recerca Hospital del Mar – IMIM, Parc de Salut Mar, Barcelona, Spain; 2 Universitat Autònoma de Barcelona, Barcelona, Spain; 3 Fundació Institut Català de Farmacologia, Barcelona, Spain; 4 Rheumatology Unit, Parc Salut Mar, Barcelona, Spain; University of Granada, Spain

## Abstract

**Background:**

The aim of this study was to describe the patterns of cannabis use and the associated benefits reported by patients with fibromyalgia (FM) who were consumers of this drug. In addition, the quality of life of FM patients who consumed cannabis was compared with FM subjects who were not cannabis users.

**Methods:**

Information on medicinal cannabis use was recorded on a specific questionnaire as well as perceived benefits of cannabis on a range of symptoms using standard 100-mm visual analogue scales (VAS). Cannabis users and non-users completed the Fibromyalgia Impact Questionnaire (FIQ), the Pittsburgh Sleep Quality Index (PSQI) and the Short Form 36 Health Survey (SF-36).

**Results:**

Twenty-eight FM patients who were cannabis users and 28 non-users were included in the study. Demographics and clinical variables were similar in both groups. Cannabis users referred different duration of drug consumption; the route of administration was smoking (54%), oral (46%) and combined (43%). The amount and frequency of cannabis use were also different among patients. After 2 hours of cannabis use, VAS scores showed a statistically significant (*p*<0.001) reduction of pain and stiffness, enhancement of relaxation, and an increase in somnolence and feeling of well being. The mental health component summary score of the SF-36 was significantly higher (*p*<0.05) in cannabis users than in non-users. No significant differences were found in the other SF-36 domains, in the FIQ and the PSQI.

**Conclusions:**

The use of cannabis was associated with beneficial effects on some FM symptoms. Further studies on the usefulness of cannabinoids in FM patients as well as cannabinoid system involvement in the pathophysiology of this condition are warranted.

## Introduction

The main complaint of patients with fibromyalgia (FM) is chronic generalized pain, although many patients suffer from concomitant symptoms, such as tiredness, morning stiffness, sleep and affective disturbances [1]. The pathophysiology of the disorder is poorly understood. Several mechanisms have been suggested including central sensitization, suppression of descending inhibitory pathways, excessive activity of glial cells, and abnormalities of neurotransmitter release [2]. In addition, blunting of the hypothalamic-pituitary-adrenal-axis (HPA-axis) and increased autonomic nervous system responsiveness have been consistently reported in FM patients. Emerging clues suggest that such dysfunction of the stress response system may be crucial in the onset of the symptoms of FM [3]. Treatment is based on the symptomatic relief of symptoms but usually modest results are obtained. The overall patient's satisfaction and the health-related quality of life are consistently poor.

Potential therapeutic uses of cannabis in different types of pain are currently extensively investigated. Data from clinical trials with synthetic and plant-based cannabinoids provide a promising approach for the management of chronic neuropathic pain of different origins [4]. Additionally, a large body of evidence currently supports the presence of cannabinoid receptors and ligands, thus an endocannabinoid neuromodulatory system appears to be involved in multiple physiological functions [5].

There is little clinical information on the effectiveness of cannabinoids in the amelioration of FM symptoms. Three clinical trials have suggested the possible benefit of cannabinoid in the management of FM [6]–[8]. Furthermore, a clinical endocannabinoid deficiency (CECD) has been hypothesized to underlie the pathophysiology of fibromyalgia, but a clear evidence to support this assumption is lacking [9].

The aim of this study was to describe the patterns of cannabis use and the associated benefits reported by patients with fibromyalgia (FM) who were consumers of this drug. In addition, the quality of life of FM patients who consumed cannabis was compared with FM subjects who were not cannabis users.

## Methods

### Patients

A cross-sectional survey was performed. Participants were identified through an advertisement from one Rheumatology Outpatients Unit, 15 associations of FM patients and 1 association of cannabis consumers, all of them located in the city of Barcelona, Spain. Recruitment began in August 2005, and the study was completed in April 2007. Patients were eligible if they were ≥18 years of age, had been diagnosed with FM according to the American College of Rheumatology criteria [1], had moderate to severe symptomatology, and were resistant to pharmacological treatment. Exclusion criteria were severe illness and history of abuse or dependence for cannabis or others psychoactive substances.

### Ethics statement

The study was approved by the local Institutional Review Board (CEIC-IMAS) and all volunteers gave their written informed consent before inclusion.

### Study procedures and evaluation

Patients were divided according their status of therapeutic cannabis use. Eligibility and exclusion criteria were checked through an accurate telephone interview. Demographic (age, gender and employment status) and clinical variables (duration of FM, number of medical consultations in the last year, associated symptoms, current pharmacological treatment, comorbid conditions, and alternative and complementary medicines) were also collected through a structured telephone interview. Patients were informed that a specific questionnaire to collect information on medicinal cannabis use will be posted to them as well as visual analogue scales (VAS) to record perceived benefits with comprehensive instructions how to fill them out.

The following variables were recorded: duration of cannabis use, previous use, cannabis derivative used (hashish or marijuana), route of administration, amount and frequency of use, supply source, physician's acknowledgement about cannabis use and changes of pharmacological treatment. Symptoms from which cannabis was used and perceived relief was recorded using 5-point Likert scale (strong relief, mild relief, not change, slight worsening, great worsening). Patients were further asked to record the perceived benefits of cannabis on a range of symptoms (pain, stiffness, relaxation, drowsiness, well-being) using 100-mm VAS scales (VAS) before and at 2 hours of cannabis consumptions. The occurrence and frequency of side effects were indicated based on a list of symptoms.

In order to compare the quality of life between users and non users of cannabis, three questionnaires were used:

The 36-item Short Form Health Survey (SF-36) is a self-administered questionnaire, validated in Spanish, in which eight dimensions of health-related quality of life are assessed: physical functioning, role-physical, bodily pain, general health, vitality, social functioning, role-emotional and mental health. Each scale is scored using norm-based methods, with higher scores indicating better health. Scores are aggregated further to produce physical and mental component summary measures of health status, using norm-based methods. The subscale scores are standardized and range from 0 to 100 with higher scores reflecting better health-related quality of life in the domain being measured [10].

The Fibromyalgia Impact Questionnaire (FIQ) is a self-administered questionnaire, validated in Spanish to assess health-related quality of life specifically in patients with fibromyalgia over the previous week. It consists of VAS and questions regarding limitations of daily living activities. The total score ranges from 0 to 80; a higher score indicates a more negative impact [11].

The Pittsburg Sleep Quality Index (PSQI) is a self-administered questionnaire, validated in Spanish, to measure the quality and patterns of sleep over the last month. It consists of 7 components that sum each other and give a total score range from 0 (no difficulties) to 21 (severe difficulties) [12].

### Statistical analysis

Data obtained from the questionnaires were analysed using the SPSS software (version 12.0.1). Comparisons were carried out using Fisher Exact tests for categorical variables and Student t test for continuous variables. The Mann-Whitney U test was used when the size of a comparison group was too small to assume normality. Statistical significance was at the 5% level and all tests were two sided.

## Results

In response to the advertisement, 70 patients contacted the researchers to inquire about the study and were screened by telephone. A total of 14 subjects, –6 cannabis users and 8 non-users–, did not meet the eligibility criteria. Therefore, 56 FM patients completed the study protocol, 28 of them were cannabis users (mainly recruited through FM association and cannabis association) and 28 were non-cannabis users (mainly recruited through FM associations and the Rheumatology Outpatients Unit of the hospital).

As shown in Table 1, there were no statistically significant differences between the cannabis users and non-users groups in any demographic or clinical variables. The most frequent comorbid diseases were also balanced between the study groups. No significant differences were observed for the percentage of patients with irritable bowel syndrome, chronic fatigue syndrome, restless legs syndrome, osteoarthritis, Sjögren's syndrome, and hypothyroidism (data not shown in Table 1). With regard to treatment based on complementary and alternative medicines, there were no significant differences between groups, neither in number (cannabis group 64%; non-users group, 75%) or modalities chosen (data not shown in Table 1).

**Table 1:** Patient characteristics*.

	Cannabis (n = 28)	Control(n = 28)	*P*-values
Age, mean ± SD	50±11.9	50±7.7	0.94^a^
Female	26 (93)	27 (96)	1.00^b^
Employment status			0.89^b^
Work disability	10 (36)	10 (36)	
Unemployment	6 (21)	5 (18)	
Currently working	5 (18)	6 (21)	
Illness dismissed	3 (11)	5 (18)	
Retired	4 (14)	2 (7)	
Disease duration (years), median (range)	5.0 (1–20)	4.0 (1–14)	0.07^c^
N° of physician visits in last year, median (range)	6.0 (0–26)	6.0 (0–46)	0.76^c^
Associated symptoms			
Widespread pain	27 (96)	28 (100)	1.00^b^
Tiredness	25 (89)	28 (100)	0.23^b^
Morning tiredness	27 (96)	27 (96)	1.00^b^
Stiffness	25 (89)	27 (96)	0.61^b^
Anxiety	24 (86)	25 (89)	1.00^b^
Sleep disturbances	24 (86)	24 (86)	1.00^b^
Headaches	22 (79)	20 (71)	0.75^b^
Mood disorders	19 (68)	22 (79)	0.54^b^
Pharmacological treatment	26 (93)	26 (93)	1.00^b^
Analgesic/Anti-inflammatory drugs	21 (75)	18 (64)	0.56^b^
Antidepressants	14 (50)	17 (61)	0.59^b^
Anxiolytics	10 (36)	10 (36)	1.00^b^
Opioids	6 (21)	11 (39)	0.24^b^
Myorelaxants	1 (4)	6 (21)	0.10^b^
Hypnotics	5 (18)	8 (29)	0.52^b^

*Except where indicated otherwise, values are n° (%);

a ^:^ t-test;

b ^: ^Fisher test;

c ^:^ U Mann-Whitney.

### Patterns of cannabis use

Of the 28 FM patients using cannabis, 11 (40%) reported a duration of cannabis use of less than one year, 9 (32%) between 1 and 3 years, and 8 (29%) more than 3 years. Only 8 patients in the cannabis group have used cannabis recreationally before the medicinal use. Cannabis derivate used in every case was marijuana. The usual methods of administration were smoking and eating, and some patients use to combine both methods. Only smokers were 11%, only eaters were 46% and those using both methods were 43%. The amount and frequency of cannabis use were diverse among patients. The most frequent doses were between 1 and 2 cigarettes each time when patients smoked and 1 spoonful each time when eating. Most of the patients (n = 12) used cannabis daily, while 5 used it 2–4 days per week, 3 used it less than twice a week and 8 patients used it only occasionally. Related amount of cannabis used in one day, 12 reported once a day, 11 reported 2–3 times a day and 3 reported more than 3 times a day. Source of supply of cannabis were from family and friends (n = 14), illicit market (n = 7), growing (n = 5) and associations (n = 2). A total of 19 patients have informed their doctor about cannabis use, and reduction of pharmacological treatment was accomplished in 19 (68%) patients as well when they started using cannabis.

### Perceived effects of cannabis use

**Figure 1:**
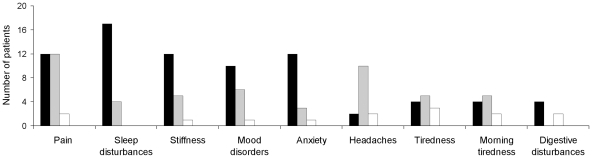
Symptoms and perceived relief reported by FM patients using cannabis. Note: Perceived relief was recorded using 5-point Likert scale (strong relief, mild relief, not change, slight worsening, great worsening). Black bars: strong relief; grey bars: mild relief; white bars: not change.

Main symptoms leading to cannabis use and perceived benefits is shown in Figure 1. Patients used cannabis not only to alleviate pain but for almost all the symptoms associated to FM, and no one reported worsening of symptoms following cannabis use. The proportion of patients who reported strong relief ranged from 81% for sleep disorders to 14% for headache.

**Figure 2:**
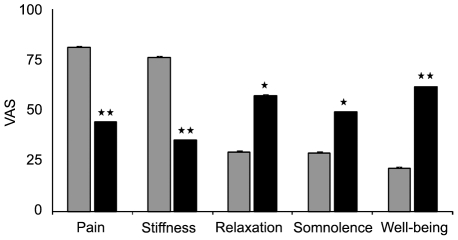
Perceived effects of cannabis self-administration. Note: Perceived benefits of cannabis recorded by patients on a range of symptoms using 100-mm VAS scales before and at 2 hours of cannabis consumptions. Grey bars: pre-cannabis; black bars: post-cannabis. * * = p<0.001; * = p<0.05.

All symptoms assessed by VAS showed statistically significant improvement following 2 hours of cannabis self-administration (Figure 2). The mean reduction of pain was 37.1 mm (*p*<0.001, t-Test) and of stiffness 40.7 mm (*p*<0.001). The change from baseline in VAS relaxation and somnolence scores also significantly increased (27.6 mm, *p*<0.05 and 20.0 mm, *p*<0.05 respectively). In addition, perception of well-being was significantly higher as compared with baseline (40.0 mm, *p*<0.001).

### Perceived side effects of cannabis use

At least one side effect was reported by 96% (n = 27) of patients. The most frequent were somnolence (n = 18), dry mouth (n = 17), sedation (n = 12), dizziness (n = 10), high (n = 9), tachycardia (n = 8), conjunctival irritation (n = 7) and hypotension (n = 6). The frequency most commonly reported were ‘sometimes’ for somnolence, sedation, dizziness, high, tachycardia and conjunctival irritation, and ‘always’ for dry mouth, sedation and hypotension. No serious adverse events occurred.

### Quality of life

The mental health component summary score of the SF-36 questionnaire was slightly but significantly higher in the cannabis group (mean (M) = 29.6±standard deviation (SD) = 8.2) than in the non-users group (M = 24.9±SD = 8.9), p<0,05, t-Test. In the physical component summary score the differences were non significant between groups (cannabis group: M = 26.29±SD = 6.7; non-users group: M = 27.34±SD = 5.8; p = 0,53, t-Test).

No differences were found either in the Fibromyalgia Impact Questionnaire (M = 65.5±SD = 11.9; M = 65.5±SD = 12.8; p = 0.36, t-Test) or in the Pittsburg Sleep Quality Index (M = 14.1±SD = 3.2; M = 14.4±SD = 3.3; p = 0.73, t-Test).

## Discussion

This observational study provides information on the patterns of cannabis use for therapeutic purposes among a group of patients with FM. Most of them were middle-aged women that did not respond to current treatment and self-administered marijuana, devoid of medical advice. Patients referred cannabis use in order to alleviate pain as well as other manifestations of FM. Significant relief of pain, stiffness, relaxation, somnolence and perception of well-being, evaluated by VAS before and 2 hours after cannabis self-administration was observed.

Although the mental health component summary score of the SF-36 questionnaire was slightly but significantly higher in the cannabis group than in the non-users group, whether these findings are clinically significant remains unclear.

The external validity of this study can be limited for some factors. The main limitation is the self-selection bias, mainly related to the fact that the majority of patients in the cannabis group were recruited from a cannabis association. It is not known how these patients are different from the ones recruited from FM associations or from the rheumatology unit. In addition the patients included in the study were all responders to cannabis self-administration. Consequently, characteristics of the patients that have used cannabis and have not obtained symptoms relief are unidentified. Others limitations were the small size of the sample and, the variability of patterns of cannabis use among FM patients.

A previous observational study of patients with chronic pain of different origins using cannabis has revealed similar results regarding symptoms relief [13]. Furthermore, significant reductions in VAS score for pain, FIQ global score and FIQ anxiety score were also seen in the first randomized controlled trial of 40 FM patients with continued pain despite the use of other medications treated with nabilone (synthetic cannabinoid agonist) during 4 weeks [7]. In a recent randomized, equivalency and crossover trial, nabilone was found to have a greater effect on sleep than amitriptyline on the ISI (Insomnia Severity Index), and was marginally better on the restfulness based on the LSEQ (Leeds Sleep Evaluation Questionnaire) [8]. These results seem to indicate a possible role of cannabinoids on the treatment of FM, although it should be confirmed in further clinical trials.

Moreover, according to hypothetical and experimental evidence, a Clinical Endocannabinoid Deficiency has been proposed to be involved on the pathophysiology of FM and other functional conditions alleviated by cannabis [9]. The participation of the endocannabinoid system in multiple physiological functions such as pain modulation, stress response system, neuroendocrine regulation and cognitive functions among others, is well known [5]. Additionally, the innovative psychoneuro-endocrinology-inmunology (PNEI) studies have shown that chronic pain may be strongly influenced by dysfunctions of the stress system and, particularly, the HPA-axis [14]. Studies have shown that the HPA- axis and the autonomic nervous system is disturbed in patients with fibromyalgia [3] and, polymorphisms of genes in the serotoninergic, dopaminergic and catecholaminergic systems may also play a role in the pathogenesis of FM [15]. Notably, these polymorphisms all affect the metabolism or transport of monoamines, compounds that have a critical role in both sensory processing and the human stress response [16]. Endocannabinoids and cannabinoid receptors are involved in the responses of animals to acute, repeated and variable stress [17] and there is good evidence that the cannabinoid receptors play a major role in modulating neurotransmitter release such as serotonin and dopamine among others [18]. However, the endocannabinoid system and its implication in stress response in humans have not been so far investigated. Because of many methodological pitfalls in life stress research, high quality studies of the role of stress in the etiopathogenesis of unexplained chronic pain syndromes, such as fibromyalgia, are scarce.

We observe significant improvement of symptoms of FM in patients using cannabis in this study although there was a variability of patterns. This information, together with evidence of clinical trials and emerging knowledge of the endocannabinoid system and the role of the stress system in the pathopysiology of FM suggest a new approach to the suffering of these patients.

The present results together with previous evidence seem to confirm the beneficial effects of cannabinoids on FM symptoms. Further studies regarding efficacy of cannabinoids in FM as well as cannabinoid and stress response system involvement in their pathophysiology are warranted.
